# Online Self-Tracking Groups to Increase Fruit and Vegetable Intake: A Small-Scale Study on Mechanisms of Group Effect on Behavior Change

**DOI:** 10.2196/jmir.6537

**Published:** 2017-03-06

**Authors:** Jingbo Meng, Wei Peng, Soo Yun Shin, Minwoong Chung

**Affiliations:** ^1^ Michigan State University Department of Communication East Lansing, MI United States; ^2^ Michigan State University Department of Media and Information East Lansing, MI United States

**Keywords:** online support group, quantified self, fruit and vegetable consumption, social comparison, similarity, social modeling

## Abstract

**Background:**

Web-based interventions with a self-tracking component have been found to be effective in promoting adults’ fruit and vegetable consumption. However, these interventions primarily focus on individual- rather than group-based self-tracking. The rise of social media technologies enables sharing and comparing self-tracking records in a group context. Therefore, we developed an online group-based self-tracking program to promote fruit and vegetable consumption.

**Objective:**

This study aims to examine (1) the effectiveness of online group-based self-tracking on fruit and vegetable consumption and (2) characteristics of online self-tracking groups that make the group more effective in promoting fruit and vegetable consumption in early young adults.

**Methods:**

During a 4-week Web-based experiment, 111 college students self-tracked their fruit and vegetable consumption either individually (ie, the control group) or in an online group characterized by a 2 (demographic similarity: demographically similar vs demographically diverse) × 2 (social modeling: incremental change vs ideal change) experimental design. Each online group consisted of one focal participant and three confederates as group members or peers, who had their demographics and fruit and vegetable consumption manipulated to create the four intervention groups. Self-reported fruit and vegetable consumption were assessed using the Food Frequency Questionnaire at baseline and after the 4-week experiment.

**Results:**

Participants who self-tracked their fruit and vegetable consumption collectively with other group members consumed more fruits and vegetables than participants who self-tracked individually (*P*=.01). The results did not show significant main effects of demographic similarity (*P*=.32) or types of social modeling (*P*=.48) in making self-tracking groups more effective in promoting fruit and vegetable consumption. However, additional analyses revealed the main effect of performance discrepancy (ie, difference in fruit and vegetable consumption between a focal participant and his/her group members during the experiment), such that participants who had a low performance discrepancy from other group members had greater fruit and vegetable consumption than participants who had a high performance discrepancy from other group members (*P*=.002). A mediation test showed that low performance discrepancy led to greater downward contrast (b=–0.78, 95% CI –2.44 to –0.15), which in turn led to greater fruit and vegetable consumption.

**Conclusions:**

Online self-tracking groups were more effective than self-tracking alone in promoting fruit and vegetable consumption for early young adults. Low performance discrepancy from other group members lead to downward contrast, which in turn increased participants’ fruit and vegetable consumption over time. The study highlighted social comparison processes in online groups that allow for sharing personal health information. Lastly, given the small scale of this study, nonsignificant results with small effect sizes might be subject to bias.

## Introduction

Extensive evidence suggests that fruit and vegetable consumption prevents obesity [1,2], reduces cardiovascular disease risk [3,4], and decreases the risk of certain cancers [5,6]. Although a growing body of literature has examined effective strategies to increase fruit and vegetable consumption in children and adolescents [7,8], young adults have been relatively understudied [9]. Early young adults aged 18 to 22 years are at an age of transitioning from parental supervision to independent living, which is an important stage of developing food patterns that will affect their future [10]. A recent report showed that adults aged 18 to 34 years consumed the least fruits and vegetables across all age groups, including adults, children, and adolescents, and their fruit and vegetable consumption had a significant decline from age groups younger than 18 years [11]. Therefore, it is worth studying effective intervention strategies to help young adults consume at least five servings of fruits and vegetables per day (ie, 5 A Day [12]).

Web-based interventions have been found to be effective in promoting fruit and vegetable consumption in adults [13], children, and adolescents [14]. However, those existing online interventions primarily focus on individual-based behavior change, missing the opportunity to leverage online groups. Approximately 62% of US adult Internet users have used health-related online groups to find experiences of others who have similar health interests [15]. The literature has suggested that social support and social influence are pathways through which such online groups may be effective for behavior change [16]. When similar people interact to increase their fruit and vegetable consumption, social support can reduce the uncertainty and costs of behavior change by providing information and companionship [17]. Social influence may also increase fruit and vegetable consumption through observational learning from behavioral models in online groups or complying with normative behavior emerged in such groups [18,19]. Moreover, approximately 70% of US adults track a health indicator, with diet and exercise routines being the most frequently monitored [18]. Given the potential of social influence in online groups and the prevalence of self-tracking behavior, online group-based programs that allow for sharing and comparing self-tracked diet may present a new intervention opportunity to increase fruit and vegetable consumption for young adults, who are characterized as a tech-savvy population [20].

This paper defines online groups as small social networks that contain three or more individuals with similar health conditions who interact via computer networks to achieve a common health goal [21,22]. Although previous research has shown effects of health-related online groups operating via social support exchanges [23,24] and social influence through social networks [25,26], few have examined the impact of groups and group characteristics on individuals’ health outcomes during the dynamic group process [27]. In the context of increasing fruit and vegetable consumption, this study aims to directly test the effectiveness of a group-based online health program and examine group characteristics (ie, demographic similarity and types of social modeling) that make a group more successful in promoting individuals’ fruit and vegetable consumption.

### Individual- Versus Group-Based Self-Tracking

Self-tracking is a way that people monitor and record specific features of their lives [28]. It allows people to reflect on the specific aspects of their health and make improvements accordingly to achieve a health goal [29]. In health behavior change literature, self-tracking is often exchangeable with self-monitoring, which refers to an intervention technique that asks people to keep a record of a target behavior [30]. Meta-analyses of behavior change interventions found that self-monitoring explained a great amount of intervention success for physical activity and healthy eating [30,31].

Self-tracking has been around for a long time. For instance, people with diabetes keep a diary about their blood sugar via glucose meters, whereas overweight people journal about their exercise activities. What is new about self-tracking is the rise of social media technologies that enable sharing and comparing personal records [32]. Social media make two modes of self-tracking convenient in practice: individual- and group-based self-tracking. In individual-based self-tracking, people collect and view information on themselves to increase self-awareness and improve their health, whereas in group-based self-tracking, people collect and share personal information with one another who self-track the same health aspect collectively [28]. Although self-tracking has been documented to have a positive effect on enhancing individual health [32], little research has compared the effectiveness of different modes of self-tracking.

This study seeks to fill this void by comparing the effectiveness of self-tracking alone or in a group wherein other group members consistently increase their fruit and vegetable consumption over time. Other group members’ fruit and vegetable consumption may exert a normative influence on the focal person. Particularly computer-mediated communication (CMC) can strengthen normative influence among members in groups [19]. According to the social identity model of deindividuation effects, interactions via computer networks accentuate group influence because of the relative anonymity that can actually facilitate individual members’ submergence in the group [33] and thus make individual members more susceptible to the influence of normative behaviors [19].

The group members not only demonstrate normative behavior of fruit and vegetable consumption, but also serve as social models to the focal person in a group. Social cognitive theory argues that observing others performing a recommended behavior is a powerful means of learning [18]. Social modeling has been frequently used in the design of dietary interventions [9,34]. Recent reviews showed that social modeling has a robust and powerful influence on food intake and choice, such that participants ate more when their modeling companions ate more [35], and that participants tended to choose the same food selected by their modeling companions [36]. Social models not only facilitate learning how to do a behavior, but also show the availability of resources in an environment for consumption (eg, access to healthy food).

Therefore, our first hypothesis is that individuals in a self-tracking group composed of members with increasing fruit and vegetable consumption over time will have greater fruit and vegetable consumption than individuals who self-track alone.

### Characteristics of Self-Tracking Groups

Groups can vary in their demographic composition, behavioral patterns, and interaction processes, which all contribute to different group dynamics and performance in achieving specific goals [37]. What are the features of a self-tracking group that make the group more effective in promoting fruit and vegetable consumption? In terms of group composition, group members who serve as social models can be similar or diverse in their demographic characteristics to the focal person. Research on social cognitive theory points out that people are more likely to learn and enact behaviors modeled by someone with whom they identify [18]. In CMC, age, gender, and ethnicity serve as important cues about the identities of group members when other social cues are filtered out [38]. People may easily use the demographic cues to categorize themselves and others into social groups [39]. Especially at the beginning of a group’s existence, when demographic characteristics are salient, individuals tend to use these characteristics in the identification process [40]. Moreover, according to the similarity-attraction principle applied in CMC [41], similarity in demographic characteristics may increase attraction to other group members, leading to a stronger group salience and susceptibility to group influence. Empirical studies have shown that demographic similarity among group members is positively associated with group performance and completing group tasks in virtual settings [42,43] in that individual group members are more motivated in high similarity groups than in diverse groups [44]. In online health groups, people consider group members as relevant references if they are demographically similar [26] and, thus, are more susceptible to their influence.

Therefore, our second hypothesis is that demographically similar online groups will have a greater effect on an individual’s fruit and vegetable consumption than demographically diverse online groups.

Although social modeling has a robust positive effect on promoting individual health behavior, very little research examines the effect of modeling the progress of behavioral change over time. Previous interventions on food consumption have focused on inducing characteristics of social models, including familiarity between the focal person and the models, models’ weight status (eg, slim, normal weight or obese), and live versus remote models [35]. Unlike those interventions in which models are simply superior in performing a recommended behavior, the models are highly likely to show different paces at which they move toward the health-related goal over time. In the context of increasing fruit and vegetable consumption, some models may make an incremental progress to reach the 5-A-Day goal (ie, incremental-change model), such as increasing one serving of fruits and vegetables per week for several weeks, whereas other models may make an ideal change to 5-A-Day immediately (ie, ideal-change model), such as increasing to eat five servings of fruits and vegetables within a week. Incremental-change models might be more effective than ideal-change models in that making a gradual progress seems more realistic and less daunting.

Particularly in online health-related groups, participants expect to meet others with similar health conditions and to compare their own situation to that of others [45]. Social comparison processes naturally occur in such online settings. The discrepancy in health behavior between group members may moderate the social comparison mechanism underlying group influence [46]. An individual’s group members, who model the recommended behavior, have to be considered encouraging and achievable to be motivating. If the group members perform much better, the individual tends to stop comparing oneself to those group members and, thus, avoid emulating the group members’ behaviors [47]. In online groups that promote fruit and vegetable consumption, performance discrepancy is the difference between the fruit and vegetable consumption of a focal person and of social models at different points of time during the process of behavioral change. On average, the focal person may have a lower performance discrepancy with the presence of incremental-change models than with the presence of ideal-change models. Incremental-change models could be more encouraging, whereas the ideal-change models might make the focal person frustrated in pursuing the health goal.

Therefore, our third hypothesis is that incremental-change models will have a greater effect on an individual’s fruit and vegetable consumption than ideal-change models in online groups.

As discussed in the rationale for the preceding hypothesis, social comparisons could be one psychological mechanism underlying the hypothesized positive effect of incremental modeling on increasing individuals’ fruit and vegetable consumption [48]. Two types of social comparisons are downward social comparisons that concern comparisons with others not doing better, and upward social comparisons that concern comparisons with others doing better [49]. Buunk and Ybema [50] argued that downward and upward comparison could be further segmented depending on whether individuals contrast themselves to or identify themselves with comparison targets. When comparing with someone worse off, individuals feel relieved and comfortable to be in a good position (ie, downward contrast), but feel anxious to be in the same situation in the future (ie, downward identification). When comparing with someone better off, individuals feel frustrated to be in a worse position (ie, upward contrast), but feel optimistic and hopeful to improve (ie, upward identification) [50]. The instances from the literature present preferences for downward contrast and upward identification because they are associated with better psychological well-being in general [45,51]. In the incremental modeling condition, social models show consistent small increases in their fruit and vegetable consumption and the focal person could perform better or worse than the models during the intervention. In the ideal modeling condition, social models quickly change their fruit and vegetable consumption and reach the ideal amount of fruit and vegetable consumption at the beginning and maintain it throughout the intervention. Then, the focal person probably performs worse than the models for most time during the intervention, although the performance discrepancy is expected to reduce over time. It is possible that both downward and upward social comparisons take place in the incremental condition: the focal person might outperform social models sometimes and when the models outperform the focal person, the low performance discrepancy may facilitate the focal person’s identification with the models. However, given the lack of previous studies on establishing the connections between types of social modeling and different social comparison processes, this study will explore the potential of this mechanism.

Therefore, our research question is will different social comparison processes mediate the positive effect of social modeling on an individual’s fruit and vegetable consumption in online groups?

## Methods

### Overview

**Figure 1:**
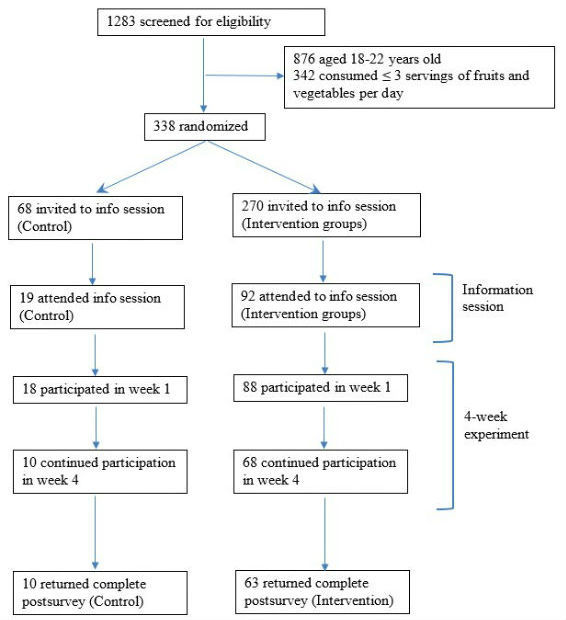
Flow diagram of the participants.

This 4-week Web-based experiment featured a 2 (demographic similarity: demographically similar vs demographically diverse) × 2 (social modeling: incremental change vs ideal change) between-subjects design plus one control group. Participants in the control group accessed the webpage and reported self-tracking of their fruit and vegetable consumption three times a week without the presence of group members. Participants in the intervention groups were asked to join a four-person group wherein every group member posted self-tracking of their fruit and vegetable consumption three times a week. Three group members in each group were confederates. Their demographics (ie, age, gender, and ethnicity) and the fruit and vegetable consumption as indicated in self-tracking posts were manipulated to create the four intervention groups. The intervention included several steps, including screening, information sessions, a 4-week Web-based experiment, and a postexperiment survey. The study design and participant flow are illustrated in Figure 1.

### Participants

Participants were recruited via messages sent to undergraduate students through the registrar’s office and two participant pools at Michigan State University, East Lansing, MI. The Human Research Protection Program at Michigan State University approved this study. Participants were first invited to take a 10-minute online survey for screening, in which they were instructed that the purpose of the survey was to assess their food consumption. Based on responses in the screening survey, the selection criteria for eligible participants for the 4-week intervention study were that the participant must (1) be age 18 to 22 years and white to achieve a clean manipulation of demographic similarity, (2) currently consume less than three servings of fruits and vegetables per day, and (3) have daily Internet access via a computer and/or a mobile phone. The screening yielded 338 eligible participants who were then sent an invitation via email to participate in the 4-week intervention. In the invitation email, they were instructed to physically attend an information session to participate in the 4-week intervention study. Then, 111 participants attended the information session and began the study. Each participant was rewarded with US $30 for completing the study.

### Procedures

Once identified as eligible, the 338 participants were randomly assigned into control and experimental conditions because the online group page needed customization for each participant based on their assigned conditions. These online group pages were created before the information sessions because participants needed to complete a few important tasks on their group pages in the information sessions. Randomization was conducted using computer-generated random digits.

A week before the experiment, 111 eligible participants attended a 30-minute information session. At the beginning of the information session, participants provided written informed consent to join the study. Then, each participant received an invitation email to join an online group page ostensibly premade based on participants’ demographic information (ie, age, gender, and ethnicity) collected from the screening survey. The starting numbers of fruit and vegetable servings of the three confederates were set up to be equal to, 0.5 above, and 0.5 below, with a mean equal to the current servings of the participant as indicated in the screening survey. After creating an online profile and joining the assigned group page, a participant was able to see the information of him- or herself and the three confederates. Participants were informed that group members’ information was obtained from the screening survey. We conducted separate information sessions for the control group and intervention groups. A premade video illustrated steps to create online profiles and join an online group page, and instructed participants to post self-tracking messages to report their fruit and vegetable consumption for 3 days each week during the 4-week experiment.

### Experiment Conditions

**Figure 2:**
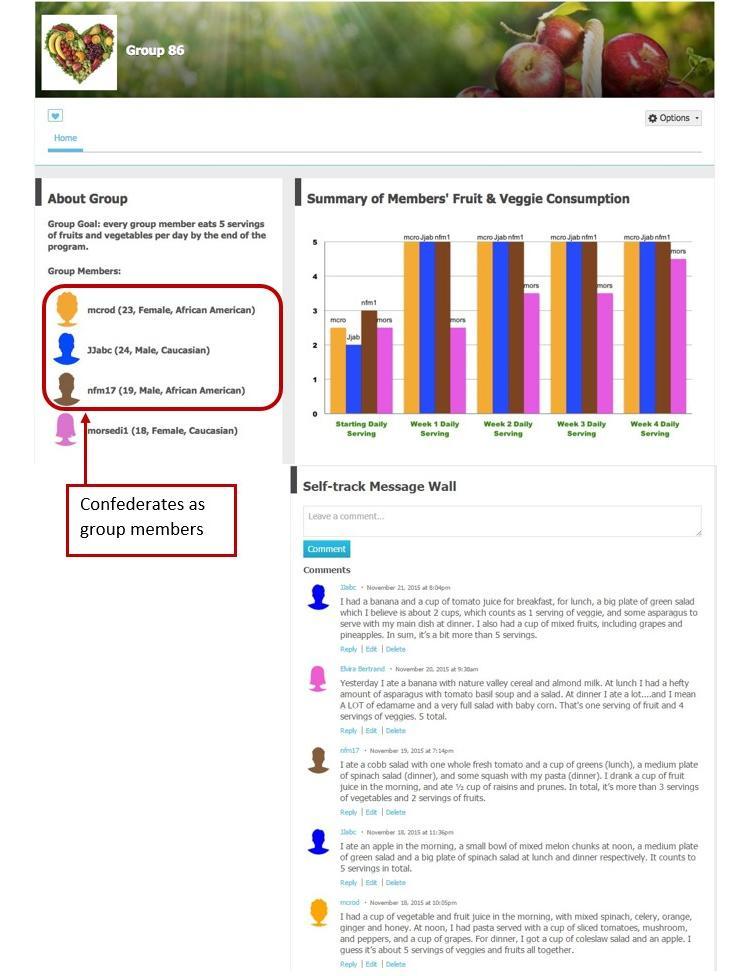
Visual example of the demographically diverse and ideal-change model intervention group page.

**Figure 3:**
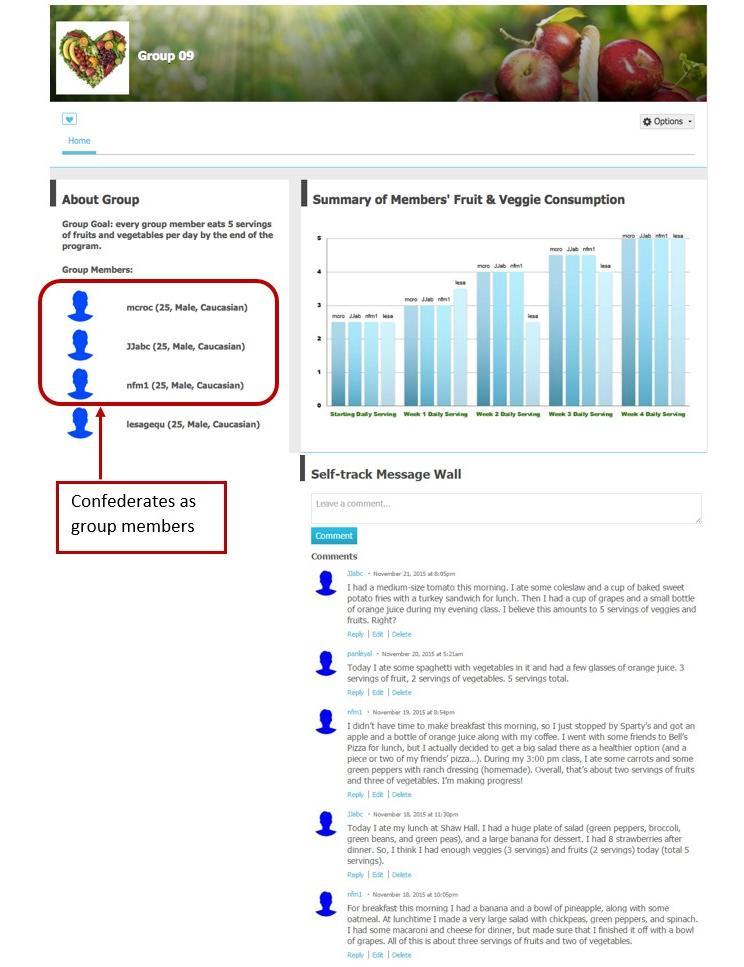
Visual example of the demographically similar and incremental-change model intervention group page.

A healthy eating community was created on Ning.com, with by-invitation-only group pages. Although the participants in the intervention groups were made to believe they were in a group with three other participants; in fact, the information of these three other participants were experimentally manipulated. Each group page consisted of three modules: (1) group goal (ie, every group member will eat five servings of fruits and vegetables per day at the end of the program) and group members’ demographics, (2) self-track message wall that allowed group members to post their fruit and vegetable consumption, and (3) a bar graph illustrating weekly summaries of fruit and vegetable consumption for each group member. Figures 2 and 3 present examples of the group pages.

#### Demographically Similar Versus Diverse Condition

Demographic similarity was manipulated by varying confederates’ displayed ages, gender, and ethnicity. In the demographically similar condition, the three confederates’ ages, gender, ethnicities were identical to the participant. In the demographically diverse condition, each confederate was different from the participant on two of the three demographic factors. For example, when a participant was an 18-year-old white female, the three confederates were a 26-year-old African-American female (ie, different age and ethnicity), an 18-year-old African-American male (ie, different ethnicity and gender), and a 25-year-old white male (ie, different age and gender).

#### Incremental-Change Versus Ideal-Change Model Condition

The type of model was manipulated by varying the confederates’ fruit and vegetable consumption over the 4-week experiment. In the incremental-change model condition, the three confederates posted prewritten messages that indicated each confederate’s average fruit and vegetable consumption to be 3, 4, 4.5, and 5 servings in the 4 weeks. In the ideal-change model condition, the three confederates posted prewritten messages that indicated each confederate’s average fruit and vegetable consumption to be five servings throughout the 4 weeks. Essentially, this manipulated the confederates’ rate of progress toward the 5-A-Day goal: incremental progress versus instant progress to achieve the ideal goal. Because we could not control a participant’s actual fruit and vegetable consumption during the 4-week experiment, the participant might sometimes outperform, underperform, or perform equally compared to others in the incremental-change condition. Textbox 1 presents examples of prewritten self-tracking messages.

**Message examples of self-tracking posts by confederates on group pages.**

5 servings: “I had a cup of coleslaw salad, one baked sweet potato and a handful of baby carrots. I ate a big orange and a banana while I was in a meeting. It’s about 5 servings of veggies and fruits all together.”

4.5 servings: “For breakfast this morning I had a bowl of oatmeal with a half-cup of dried apricots mixed in and a glass of orange juice. At lunch I had a cup of chicken soup and a medium-sized salad, with an apple for an afternoon snack. I then ate two slices of pizza and about three carrot sticks for dinner. This should make three servings of fruits and one-and-a-half servings of vegetables. So, 4.5 total. Not bad today.”

4 servings: “I ate a cobb salad with one fresh tomato and two cups of greens (lunch), and I drank a cup of fruit juice in the morning, and ate ½ cup of raisins and prunes. It’s about 2 servings of veggies and 2 serving of fruit.”

3 servings: “I had a ½ cantaloupe today. I love melons. For veggies, I had a cup of coleslaw at noon. It’s about 1 serving of veggies and 2 servings of fruits.”

### Measurement

The primary outcome fruit and vegetable consumption was measured using the Food Frequency Questionnaire (FFQ) [52]. The FFQ asked about the amount and the frequency of consumption of 23 kinds of vegetables (eg, string beans, green beans, cooked greens such as spinach, mustard greens) and 12 kinds of fruits (eg, nectarines, plums, watermelon, honeydew) over the past 4 weeks. For the amount of consumption, participants could select the serving size (small, medium, large). The pictorial examples of different serving sizes were provided along with the questions. For the frequency of consumption, participants were asked to rate on an 8-point scale: (1=never or less than once per month, 8=2 or more per day).

Social comparison processes were measured by four different indexes each consisting of three items [49]. Participants were asked to rate their feelings on a 5-point scale (1=not at all, 5=very much) when reading other group members’ self-tracking messages and weekly summaries of fruit and vegetable consumption. One index measured downward contrast (eg, “...I am happy that I am doing well myself,” Cronbach alpha=.92; mean 3.14, SD 1.04), one index measured upward contrast (eg, “...I feel frustrated about my own situation,” Cronbach alpha=.91; mean 2.12, SD 1.08), the other two indexes measured upward identification (eg, “...I realize that it is possible to improve,” Cronbach alpha=.77; mean 3.70, SD 0.93) and downward identification (eg “...I fear that my future will be similar,” Cronbach alpha=.83; mean 1.77, SD 0.86).

In addition to age and gender, participants’ baseline fruit and vegetable consumption was measured in the screening survey. Self-reported height and weight were used to compute body mass index (BMI) scores. Enrollment in a campus meal plan (1=yes, 0=no) was measured because meal plan participants tended to have the recommended daily fruit and vegetable consumption [53].

### Analytic Plan

First, a series of independent-sample *t* tests and chi-square tests were conducted to check the equivalence in terms of age, gender composition, BMI, etc, between participants who attended the information session and those who did not attend, between participants who began the study (ie, attended the information session) but dropped out later and those who completed the study, and between participants assigned into the different experimental conditions. The same methods of analyses were used to check the success of our manipulations.

To test our hypotheses, using intention-to-treat analysis [54], differences in fruit and vegetable consumption after the intervention were assessed with linear mixed-effects models, including conditions and time (ie, the time at which fruit and vegetable consumption was measured: baseline vs posttest) as independent variables, and a random intercept to account for missing data. Specifically, to test our first hypothesis that individuals in a self-tracking group composed of members with fruit and vegetable consumption that increases over time will have greater fruit and vegetable consumption than individuals who self-track alone, the study condition (control vs intervention condition) was entered together with time as the independent variables. To test the hypotheses that demographically similar online groups and incremental-change models will have a greater effect on an individual’s fruit and vegetable consumption than demographically diverse online groups and ideal-change models, demographic similarity (demographically similar vs diverse) and types of social modeling (incremental change vs ideal change) together with time were entered as the independent variables. The time factor was introduced to include dropouts in the analysis and to have a grouping factor for mixed-effects analysis. As random effects, we allowed each participant to have differing intercepts. The *P* values for testing statistical significance of each independent variable were obtained by *F* tests with the Kenward-Roger approximation [55]. These analyses were conducted using the R package “Ime4” [56]. Then, our research question was tested in a parallel mediation model using bootstrapping [57]. Bootstrapping has been recommended for testing indirect effects on small to moderate samples because the Baron and Kenny method [58] is one of the least powerful approaches to testing mediation due to its reliance on a number of inferential procedures [59].

## Results

### Sample Characteristics

A total of 73 participants completed the 4-week intervention and the postsurvey. Among them, 49 were female, and the mean age was 19.86 (SD 1.65) years. The 73 participants consumed a mean 2.03 (SD 0.72) servings per day at baseline and a mean 3.05 (SD 2.01) servings per day after the 4-week experiment. To compare eligible participants who attended and who did not attend the information session. The results show that they did not differ on age (*t*_333_=–1.61, *P*=.11), baseline fruit and vegetable consumption (*t*_333_*=* 1.29, *P*=.19), or gender (χ^2^_1_=0.3, *P*=.66), although participants who attended the information session reported more interest on a 5-point scale (mean 2.36, SD 1.25) in participating in an online support group for healthy eating than those who did not attend the information session (mean 1.94, SD 1.13; *t*_333_=2.49, *P*=.01).

For the 111 participants who attended the information session, 73 (65.8%) completed the 4-week intervention and the postsurvey. Analyses showed that participants who began the study but dropped out did not differ from those who completed the study on age (*t*_109_=1.53, *P*=.13), gender (χ^2^_1_=0.1, *P*=.81), BMI (*t*_109_=–0.69, *P*=.49), baseline fruit and vegetable consumption (*t*_109_=0.24, *P*=.81), or interest in participating in an online support group for heathy eating (*t*_109_=1.52, *P*=.13).

Table 1 presents descriptive statistics of the samples split by conditions, including baseline fruit and vegetable consumption, BMI, age, gender distribution, and completion rate. Participants across conditions did not significantly differ on any of these characteristics.

**Table 1:** Baseline fruit and vegetable consumption, BMI, age, gender, meal plan, and completion rate for the intervention conditions (N=73).

Sample characteristics	Condition					Total sample (N = 73)	*F*_4,68_	χ^2^_4_	*P*
	Similar/incremental (n=17)	Diverse/incremental (n=14)	Similar/ideal (n=16)	Diverse/ideal (n=16)	Control (n=10)				
Baseline fruit and vegetable consumption, mean (SD)	1.92 (0.76)	2.25 (0.67)	2.17 (0.66)	2.07 (0.78)	1.94 (0.76)	2.04 (0.73)	0.93		.45
BMI (kg/m^2^), mean (SD)	23.05 (3.32)	22.06 (2.26)	22.43 (2.15)	22.96 (3.60)	21.47 (2.91)	22.74 (2.98)	1.55		.20
Age (years), mean (SD)	19.76 (1.79)	19.17 (1.53)	19.75 (1.07)	20.27 (1.83)	20.40 (2.01)	19.86 (1.65)	1.06		.39
Gender (female), n (%)	13 (77)	11 (79)	10 (63)	9 (56)	6 (60)	49 (67)		2.75	.60
Meal plan (yes), n (%)	12 (71)	9 (64)	11 (69)	11 (69)	7 (70)	50 (69)		2.59	.63
Completion rate, n (%)^a^	24 (71)	21 (67)	23 (70)	24 (67)	19 (53)	111 (65.8)		3.53	.47

^a^ Completion rate was calculated using the number of participants who completed the postintervention survey divided by the number of participants who attended the information session.

### Manipulation Check

To check whether the manipulation for group demographic similarity was successful, in the postexperiment survey we asked participants to indicate which of the following statements best described their group members: “We are diverse in terms of age, gender, and ethnicity,” (coded as 0) and “We are similar in terms of age, gender, and ethnicity” (coded as 1). A chi-square test showed a significant difference between demographically similar groups (mean 1.81, SD 0.40) and diverse groups (mean 1.00, SD 0.01; χ^2^_1_=11.2, *P*<.001).

To check whether the manipulation for incremental- versus ideal-change models was successful, in a pilot test with 10 participants we asked them to indicate whether other group members’ fruit and vegetable consumption increased gradually (coded as 0) or stayed the same (coded as 1). A chi-square test showed that participants in the incremental modeling condition were significantly more likely to choose 0, whereas participants in the ideal modeling condition were more likely to choose 1 (χ^2^_1_=10.0, *P*=.008).

We also included two indirect manipulation checks for the incremental- versus ideal-change models: actual and perceived performance discrepancy. Actual performance discrepancy was calculated based on participants’ wall posts on the group pages. It was the mean difference between manipulated serving sizes of social models and actual serving sizes reported by participants in their self-tracking messages across the first 3 weeks during the 4-week intervention. Perceived performance discrepancy was measured in the postexperiment survey by asking participants to rate whether other group members performed worse than (=1), similar to (=2), a little bit better (=3), or much better (=4) than them in achieving 5 A Day. The *t* tests revealed no significant difference in actual performance discrepancy between the incremental-change (mean 0.82, SD 0.75) and ideal-change models (mean 1.18, SD 1.04; *t*_71_=–1.48, *P*=.15), or in perceived performance discrepancy between the incremental-change (mean 3.35, SD 0.79) and ideal-change models (mean 3.29, SD 0.69; *t*_71_=–0.34, *P*=.74). Therefore, incremental versus ideal modeling did not necessarily induce low versus high performance discrepancy.

### Individual- Versus Group-Based Self-Tracking

The results showed that participants assigned to intervention groups (ie, group-based self-tracking) consumed more fruits and vegetables than those assigned to the control group (ie, individual-based self-tracking; beta=.20, *F*_1,88.25_=6.83, *P*=.01). Participants who self-tracked their fruit and vegetable consumption collectively with other group members (mean 3.37, SD 2.01) consumed more fruits and vegetables than participants who self-tracked their fruit and vegetable consumption alone (mean 1.37, SD 1.44).

### Group-Based Self-Tracking

Demographic similarity did not show a significant main effect on fruit and vegetable consumption (beta=–.06, *F*_1,76.92_=1.02, *P*=.32). Types of social modeling did not show a significant effect either (beta=.04, *F*_1,75.88_=0.50, *P*=.48). There was no interaction effect between demographic similarity and types of social modeling (*P*=.46). In other words, there was no difference between the demographically similar groups (mean 3.40, SD 2.55) and the demographically diverse groups (mean 3.19, SD 1.45) in fruit and vegetable consumption after the 4-week experiment. Similarly, no difference was found between the groups with incremental-change models (mean 3.58, SD 2.30) and the groups with ideal-change models (mean 3.18, SD 1.70) in terms of fruit and vegetable consumption. Moreover, the results showed a significant main effect of time (beta=.34, *F*_1,77.52_=24.82, *P*<.001), such that participants’ fruit and vegetable consumption increased over the course of the intervention. However, there were no significant interaction effects between time and the experimental conditions.

### Post Hoc Analysis: Performance Discrepancy

Our indirect manipulation check demonstrated that the incremental-change and ideal-change models did not necessarily create the performance discrepancy as we expected; therefore, we explored the effect of performance discrepancy as the independent variable in the post hoc analysis. Using the mean of actual performance discrepancy (mean 0.96, SD 0.92), participants were split into low (coded as 0, n=32) versus high (coded as 1, n=31) actual performance discrepancy groups. A linear mixed-effects model with a random intercept to account for missing data was used to assess the difference in fruit and vegetable consumption after the intervention, including performance discrepancy, demographic similarity, and time as the independent variables. The results revealed a significant main effect of actual performance discrepancy on participants’ fruit and vegetable consumption at the end of the 4-week experiment (beta=–.29, *F*_1,56_=12.64, *P*=.002). Specifically, participants who had a low performance discrepancy from other group members had greater fruit and vegetable consumption (mean 4.11, SD 2.28) than participants who had a high performance discrepancy from other group members (mean 2.56, SD 1.32). Using a similar analytic method, an additional analysis was conducted by using actual values of performance discrepancy (ie, without dichotomizing performance discrepancy) as one of the independent variables. The result showed the same result, such that performance discrepancy had a negative effect on fruit and vegetable consumption (beta=–.35, *P*=.001). Given the significant main effect of actual performance discrepancy, a series of post hoc analyses were conducted to test if different social comparison processes mediated the effect of performance discrepancy on fruit and vegetable consumption. Indirect effects were tested in a parallel mediation model using bootstrapping [57]. The existence of an indirect effect was determined by the following two criteria. First, the total effect should be statistically significant, and the direct effect should become statistically nonsignificant. Second, the indirect effect should be statistically significant [57]. As presented in Table 2, the indirect effect through downward contrast was significant with a coefficient of –0.78 (95% CI –2.44 to –0.15). It showed that low performance discrepancy led to greater downward contrast, which in turn led to greater fruit and vegetable consumption. Therefore, downward contrast mediated the relationship between performance discrepancy and fruit and vegetable consumption.

**Table 2:** Tests of indirect effect of performance discrepancy on fruit and vegetable consumption through social comparisons.

Mediation tests		b (SE)	*P*
**Performance discrepancy to social comparisons**			
	Performance discrepancy to upward contrast	0.64 (0.26)	.03
	Performance discrepancy to downward contrast	–0.85 (0.25)	.001
	Performance discrepancy to upward identification	–0.45 (0.24)	.09
	Performance discrepancy to downward identification	0.25 (0.22)	.12
**Social comparisons to fruit and vegetable consumption**			
	Upward contrast to fruit and vegetable consumption	0.58 (0.29)	.07
	Downward contrast to fruit and vegetable consumption	0.84 (0.29)	.01
	Upward identification to fruit and vegetable consumption	–0.37 (0.29)	.67
	Downward identification to fruit and vegetable consumption	–0.20 (0.32)	.58
**Total effect of performance discrepancy on fruit and vegetable consumption**		–1.46 (0.49)	.01
**Direct effect of performance discrepancy on fruit and vegetable consumption**		–1.03 (0.53)	.08
**Indirect effects of performance discrepancy on fruit and vegetable consumption through social comparisons**			
	Through upward contrast (bias-corrected 95% CI –0.12 to 1.53)	0.37 (0.37)	
	Through downward contrast (bias-corrected 95% CI –2.44 to –0.15)	–0.78 (0.39)	
	Through upward identification (bias-corrected 95% CI –0.21 to 0.47)	0.05 (0.15)	
	Through downward identification (bias-corrected 95% CI –0.77 to 0.13)	0.00 (0.16)	

## Discussion

This study built an online group-based program that allows for self-tracking dietary behavior for young adults. A significantly greater fruit and vegetable consumption was evident when participants self-tracked in groups wherein other group members showed consistent increases in fruit and vegetable consumption than when participants self-tracked alone. Therefore, our first hypothesis was supported. The finding suggests the effectiveness of using online self-tracking groups with social models for young adults. Although self-tracking helps to increase self-awareness of one’s fruit and vegetable consumption [28], people need a larger context, such as a group environment, where they could observe and compare with others’ performances to make more significant increases in fruit and vegetable consumption.

### Theoretical Implications

The lack of differences between demographically similar and diverse groups in increasing fruit and vegetable consumption revealed that demographic similarity was not a determining group characteristic that made a self-tracking group more successful in behavior change. Social cognitive theory states that social learning and behavior change are more likely to take place when a person perceives a strong identification with a model [18]. In this study, young adults may not necessarily identify more with others sharing similar demographic characteristics because young adults live in a multiracial and multicultural society where demographic diversity is natural in everyday encounters [60,61]. Moreover, participants were put in groups with anonymous strangers. Although the manipulation of demographic similarity may foster some degree of identification with group members, groups consisting of already-known people may present a completely different level of identification, likely to be stronger than what we found in this study. Future research is encouraged to study different group compositions such that group members have preexisting connections, and to assess the effect of speed at which models progress toward a health goal in a naturalistic experiment.

An alternative explanation is that eating five servings of fruits and vegetables per day is a healthy behavior that applies to all [62], regardless of age, gender, or ethnicity. Therefore, demographic characteristics of the models may not play a critical role here. In addition, although participants in self-tracking groups collectively worked toward the 5-A-Day goal, they were not interdependent on one another to achieve the goal. Group literature that showed significant effects of demographic similarity on performance often involved higher levels of interdependency and interactions among group members [40]. For members who work coactively rather than interdependently, the effect of demographic similarity is probably minimal.

Incremental-change models did not show an advantage in increasing individuals’ fruit and vegetable consumption when compared with ideal-change models. The finding suggested that the speed at which social models achieved the health goal did not affect individuals’ behavior change. The manipulation check showed that, contrary to our hypothesis, incremental-change models did not necessarily lead to a lower actual or perceived performance discrepancy than ideal-change models. The participants’ actual performance discrepancy was consistent with their perceived performance discrepancy, and that actual performance discrepancy had a significant effect on increasing individuals’ fruit and vegetable consumption. Taken together, what makes an online group more effective is not the models’ progress of behavior change but the amount of difference from the models’ performance in behavior change. Participants with a low performance discrepancy from models in the same online groups consumed more fruits and vegetables than participants with a high performance discrepancy from models. High performance discrepancy may make the focal person believe that he or she can never match or exceed perpetually superior group members [63]. High performance discrepancy then may weaken individuals’ efficacy to increase their fruit and vegetable consumption persistently over time. Interestingly, it may also be that consistent upward or downward comparisons are less effective in behavior change because they present less motivating or challenging scenarios than an upward-downward combined comparison. Participants with a low performance discrepancy from models sometimes outperformed, underperformed, or equally performed compared to models (discrepancy ranged from –0.48 to 0.96 servings), whereas participants with a high performance discrepancy always underperformed compared to models (discrepancy ranged from 1.00 to 3.29 servings). Future research should test this speculation and increase our understanding of effective social comparison mechanisms for behavior change.

In addition, our post hoc analysis revealed that downward contrast mediated the effect of performance discrepancy on fruit and vegetable consumption outcomes. When comparing with group members who performed worse or similarly now and then, the focal person would feel good and relieved that they were doing well. However, upward identification was not a significant mediator. When comparing with group members who performed better, upward identification was the mechanism that could lead to positive behavior change but this was not observed in this study. Therefore, in online groups, in addition to matching group members with similar performance (ie, sometimes worse, sometimes a bit better) to facilitate downward contrast, it is critical to activate upward identification so that participants can emulate better models.

### Practical Implications

This study provides several insights into the design of effective online groups to promote fruit and vegetable consumption for young adults. With the rapid growth of various self-tracking technologies [29], virtual connections among self-trackers allow for sharing of personal health information. Collective self-tracking in a group is more effective than self-tracking alone, when group members show positive progress toward the health goal. Existing self-tracking mobile apps and online communities (eg, MyFitnessPal, FatSecret) may leverage this insight to virtually connect self-trackers into small groups. For people who need to increase their fruit and vegetable consumption, such as patients with diabetic or cardiovascular diseases, health care providers may want to prescribe beyond self-tracking practice and encourage them to get connected with other self-trackers via online or offline support groups or via mobile networks.

Moreover, previous studies have found that people in online health social networks tend to connect with others with similar demographic backgrounds and similar progress toward a shared health goal [26]. This study found that it was the similarity in health progress rather than similarity in demographic background that made online groups more effective in promoting fruit and vegetable consumption behavior. Therefore, in creating online groups or online social networks for increasing people’s fruit and vegetable consumption, algorithms may be developed to recommend teaming up with others who have similar health progress toward the goal.

### Limitations

There were a few limitations in this study, beyond the reasonable attrition. First, for the 338 participants who were eligible and invited to participate in the 4-week intervention, only 111 (32.8%) participants attended the information session of the study. The reason for the loss of eligible participants in the information session was that student participants recruited from the registrar’s office and participant pools typically had choices to participate in other studies. Student participants tended to choose studies that cost minimal effort, such as an online survey. Our study involved physical attendance to the information session and a 4-week intervention, which was less attractive to student participants. However, our analysis showed that there were not any significant differences between eligible participants who attended and who did not attend the information session in terms of age, gender, and baseline fruit and vegetable consumption.

Second, in post hoc power analyses, we had a 74% observed power to detect a significant difference between the control versus intervention groups with Cohen *d*=0.63, an 80% power to detect a significant main effect of performance discrepancy on postintervention fruit and vegetable consumption (post hoc analysis) with Cohen *d*=0.61. However, we only had a 13% power to detect a significant main effect of social modeling on postintervention fruit and vegetable consumption with Cohen *d*=0.12, and a 10% power for demographic similarity with Cohen’s *d*=0.08. Therefore, the small sample size might have contributed to the null findings for our hypotheses that demographically similar online groups and incremental-change models will have a greater effect on an individual’s fruit and vegetable consumption than demographically diverse online groups and ideal-change models, with very small effect sizes. Similarly, group equivalence tests in sample characteristics may be biased by the small sample size in each condition. Readers are cautioned to interpret these results with the small sample size and lack of power in mind.

Third, this study was a short-term behavior change (ie, 4 weeks). We have no knowledge whether fruit and vegetable consumption would continue to increase or remain at five servings. Future studies should examine the potential long-term effect of online groups for changing fruit and vegetable intake. Lastly, the self-tracking method used in this intervention was traditional self-reported food journaling, which imposes a high burden on participants [64]. Recent automated food recognition technologies rely on wearable cameras or phones to capture food photos and leverage computer vision techniques to analyze food ingredients [65,66]. Another alternative method is to use an in-the-moment photo as a lightweight food journal to reduce user effort [67]. Future research should consider these more advanced self-tracking methods to reduce participation attrition as well as increase the accuracy and usefulness of self-tracked information.

### Conclusion

This study is one of the first attempts to test the effects of online self-tracking groups in increasing fruit and vegetable consumption for young adults. The 4-week experiment showed that online self-tracking groups with models consistently increasing their fruit and vegetable consumption were more effective than self-tracking alone in promoting fruit and vegetable consumption. We also found that low performance discrepancy from models would lead to downward contrast, which in turn increases participants’ fruit and vegetable consumption over time. The study highlighted social comparison processes in online groups that allow for sharing personal health information.

## Abbreviations

BMI: body mass index
CMC: computer-mediated communication
FFQ: Food Frequency Questionnaire

## References

[1] Casazza K, Fontaine KR, Astrup A, Birch LL, Brown AW, Bohan Brown MM, Durant N, Dutton G, Foster EM, Heymsfield SB, McIver K, Mehta T, Menachemi N, Newby PK, Pate R, Rolls BJ, Sen B, Smith DL, Thomas DM, Allison DB (2013). Myths, presumptions, and facts about obesity. N Engl J Med.

[2] Rolls BJ, Ello-Martin JA, Tohill BC (2004). What can intervention studies tell us about the relationship between fruit and vegetable consumption and weight management?. Nutr Rev.

[3] Bazzano LA, He J, Ogden LG, Loria CM, Vupputuri S, Myers L, Whelton PK (2002). Fruit and vegetable intake and risk of cardiovascular disease in US adults: the first National Health and Nutrition Examination Survey Epidemiologic Follow-up Study. Am J Clin Nutr.

[4] Dauchet L, Amouyel P, Hercberg S, Dallongeville J (2006). Fruit and vegetable consumption and risk of coronary heart disease: a meta-analysis of cohort studies. J Nutr.

[5] Steinmetz KA, Potter JD (1996). Vegetables, fruit, and cancer prevention: a review. J Am Diet Assoc.

[6] Turati F, Rossi M, Pelucchi C, Levi F, La VC (2015). Fruit and vegetables and cancer risk: a review of southern European studies. Br J Nutr.

[7] Mathias KC, Rolls BJ, Birch LL, Kral TV, Hanna EL, Davey A, Fisher JO (2012). Serving larger portions of fruits and vegetables together at dinner promotes intake of both foods among young children. J Acad Nutr Diet.

[8] Thompson D, Bhatt R, Vazquez I, Cullen KW, Baranowski J, Baranowski T, Liu Y (2015). Creating action plans in a serious video game increases and maintains child fruit-vegetable intake: a randomized controlled trial. Int J Behav Nutr Phys Act.

[9] Peng W (2009). Design and evaluation of a computer game to promote a healthy diet for young adults. Health Commun.

[10] Richards A, Kattelmann KK, Ren C (2006). Motivating 18- to 24-year-olds to increase their fruit and vegetable consumption. J Am Diet Assoc.

[11] Produce for Better Health Foundation (2015). State of the Plate: 2015 Study on America's Consumption of Fruit & Vegetables.

[12] Casagrande SS, Wang Y, Anderson C, Gary TL (2007). Have Americans increased their fruit and vegetable intake? The trends between 1988 and 2002. Am J Prev Med.

[13] Buller DB, Woodall WG, Zimmerman DE, Slater MD, Heimendinger J, Waters E, Hines JM, Starling R, Hau B, Burris-Woodall P, Davis GS, Saba L, Cutter GR (2008). Randomized trial on the 5 a day, the Rio Grande Way Website, a web-based program to improve fruit and vegetable consumption in rural communities. J Health Commun.

[14] Hamel LM, Robbins LB (2013). Computer- and web-based interventions to promote healthy eating among children and adolescents: a systematic review. J Adv Nurs.

[15] Fox S (2011). The Social Life of Health Information.

[16] Berkman LF, Glass T, Brissette I, Seeman TE (2000). From social integration to health: Durkheim in the new millennium. Soc Sci Med.

[17] Cavallo DN, Brown JD, Tate DF, DeVellis RF, Zimmer C, Ammerman AS (2014). The role of companionship, esteem, and informational support in explaining physical activity among young women in an online social network intervention. J Behav Med.

[18] Bandura A (1986). Social Foundations of Thought and Action: A Social Cognitive Theory.

[19] Postmes T, Spears R, Lea M (1998). Breaching or building social boundaries?: SIDE-effects of computer-mediated communication. Commun Res.

[20] Duggan M, Ellison NB, Lampe C, Lenhart A, Madden M (2015). Pew Research Center.

[21] Centola D (2013). Social media and the science of health behavior. Circulation.

[22] Maloney-Krichmar D, Preece J (2005). A multilevel analysis of sociability, usability, and community dynamics in an online health community. ACM Trans Comput-Hum Interact.

[23] Meng J, Martinez L, Holmstrom A, Chung M, Cox J (2016). Research on social networking sites and social support from 2004 to 2015: a narrative review and directions for future research. Cyberpsychol Behav Soc Netw.

[24] Meng J, Chung M, Cox J (2016). Linking network structure to support messages: effects of brokerage and closure on received social support. J Commun.

[25] Centola D (2010). The spread of behavior in an online social network experiment. Science.

[26] Meng J (2016). Your health buddies matter: preferential selection and social influence on weight management in an online health social network. Health Commun.

[27] Kreps GL, Neuhauser L (2010). New directions in eHealth communication: opportunities and challenges. Patient Educ Couns.

[28] Lupton D (2014). Self-tracking cultures: towards a sociology of personal informatics. Australian Computer-Human Interaction Conference on Designing Futures: The Future of Design.

[29] Swan M (2009). Emerging patient-driven health care models: an examination of health social networks, consumer personalized medicine and quantified self-tracking. Int J Environ Res Public Health.

[30] Dusseldorp E, van Genugten L, van Buuren S, Verheijden MW, van Empelen P (2014). Combinations of techniques that effectively change health behavior: evidence from Meta-CART analysis. Health Psychol.

[31] Michie S, Abraham C, Whittington C, McAteer J, Gupta S (2009). Effective techniques in healthy eating and physical activity interventions: a meta-regression. Health Psychol.

[32] Wiederhold BK (2012). Self-tracking: better medicine through pattern recognition. Cyberpsychol Behav Soc Netw.

[33] Lea M, Spears R (1991). Computer-mediated communication, de-individuation and group decision-making. Int J Man Mach Stud.

[34] Baranowski T, Cullen KW, Nicklas T, Thompson D, Baranowski J (2003). Are current health behavioral change models helpful in guiding prevention of weight gain efforts?. Obes Res.

[35] Vartanian LR, Spanos S, Herman CP, Polivy J (2015). Modeling of food intake: a meta-analytic review. Soc Influ.

[36] Cruwys T, Bevelander KE, Hermans RC (2015). Social modeling of eating: a review of when and why social influence affects food intake and choice. Appetite.

[37] Poole M, Hollingshead A (2005). Theories of Small Groups: Interdisciplinary Perspectives.

[38] Walther J, Parks M, Knapp ML, Daly JA (2002). Cues filtered out, cues filtered in: computer-mediated communication and relationships. Handbook of Interpersonal Communication.

[39] Turner J (1987). Rediscovering the Social Group: A Self-Categorization Theory.

[40] Price KH, Harrison DA, Gavin JH, Florey AT (2002). Time, teams, and task performance: changing effects of surface- and deep-level diversity on group functioning. Acad Manage J.

[41] Nass C, Lee KM (2001). Does computer-synthesized speech manifest personality? Experimental tests of recognition, similarity-attraction, and consistency-attraction. J Exp Psychol Appl.

[42] Horwitz SK, Horwitz IB (2007). The effects of team diversity on team outcomes: a meta-analytic review of team demography. J Manage.

[43] van der Land SF, Schouten AP, Feldberg F, Huysman M, van den Hooff B (2014). Does Avatar appearance matter? How team visual similarity and member-Avatar similarity influence virtual team performance. Hum Commun Res.

[44] Watson WE, Johnson L, Kumar K, Critelli J (1998). Process gain and process loss: comparing interpersonal processes and performance of culturally diverse and non-diverse teams across time. Int J Intercult Rel.

[45] Batenburg A, Das E (2015). Virtual support communities and psychological well-being: the role of optimistic and pessimistic social comparison strategies. J Comput-Mediat Comm.

[46] Kerr NL, Messé LA, Seok D, Sambolec EJ, Lount RB, Park ES (2007). Psychological mechanisms underlying the Köhler motivation gain. Pers Soc Psychol Bull.

[47] Locke E, Golembiewski RT (2001). Motivation by goal setting. Handbook of Organizational Behavior.

[48] Festinger L (1954). A theory of social comparison processes. Hum Relat.

[49] Van der Zee K, Buunk B, Sanderman R, Botke G, van den Bergh F (2000). Social comparison and coping with cancer treatment. Pers Indivl Differ.

[50] Buunk B, Ybema J, Buunk B, Gibbons FX (1997). Social comparisons and occupational stress: the identification-contrast model. Health, Coping and Well-being: Perspectives from Social Comparison Theory.

[51] Dibb B, Yardley L (2006). How does social comparison within a self-help group influence adjustment to chronic illness? A longitudinal study. Soc Sci Med.

[52] Thompson FE, Byers T (1994). Dietary assessment resource manual. J Nutr.

[53] Brown LB, Dresen RK, Eggett DL (2005). College students can benefit by participating in a prepaid meal plan. J Am Diet Assoc.

[54] Gupta SK (2011). Intention-to-treat concept: a review. Perspect Clin Res.

[55] Kenward MG, Roger JH (1997). Small sample inference for fixed effects from restricted maximum likelihood. Biometrics.

[56] Bates D, Mächler M, Bolker B, Walker S (2015). Fitting linear mixed-effects models using lme4. J Stat Soft.

[57] Hayes A (2013). Introduction to Mediation, Moderation, and Conditional Process Analysis: A Regression-Based Approach.

[58] Baron RM, Kenny DA (1986). The moderator-mediator variable distinction in social psychological research: conceptual, strategic, and statistical considerations. J Pers Soc Psychol.

[59] Mackinnon DP, Lockwood CM, Williams J (2004). Confidence limits for the indirect effect: distribution of the product and resampling methods. Multivariate Behav Res.

[60] Parker K, Morin R, Horowitz J, Lopez M, Rohal M (2015). Pew Research Center.

[61] (2014). United States Census Bureau.

[62] Centers for Disease Control and Prevention (2005). 5 A Day Works!.

[63] Kerr NL, Forlenza ST, Irwin BC, Feltz DL (2013). “… been down so long …”: Perpetual vs intermittent inferiority and the Köhler group motivation gain in exercise groups. Group Dyn-Theor Res.

[64] Baranowski T, Willett W (2012). 24-hour recall and diet record methods. Nutritional Epidemiology. Third edition.

[65] Bettadapura V, Thomaz E, Parnami A, Abowd G, Essa I (2015). Leveraging context to support automated food recognition in restaurants. Proceedings of WACV 2015: IEEE Winter Conference on Applications of Computer Vision.

[66] Merler M, Wu H, Uceda-Sosa R, Nguyen Q, Smith J (2016). Snap, Eat, RepEat: a food recognition engine for dietary logging. Proceedings of the 2nd International Workshop on Multimedia Assisted Dietary Management.

[67] Cordeiro F, Bales E, Cherry E, Fogarty J (2015). Rethinking the mobile food journal: Exploring opportunities for lightweight photo-based capture. Proceedings of the 33rd Annual ACM Conference on Human Factors in Computing Systems.

